# Clinical characteristics and microbiota analysis of 44 patients with granulomatous mastitis

**DOI:** 10.3389/fmicb.2023.1175206

**Published:** 2023-04-17

**Authors:** Wen Chen, Dongxiao Zhang, Yifei Zeng, Jianchun Cui, Jiale Yu, Junyue Wang, Shuqi Li, Qiao Huang, Khattak Mazher Mansoor

**Affiliations:** ^1^Breast Department, Beijing Hospital of Traditional Chinese Medicine, Capital Medical University, Beijing, China; ^2^Beijing University of Chinese Medicine, Beijing, China; ^3^Department of Thyroid and Breast Surgery, Liaoning Provincial People's Hospital, People's Hospital of China Medical University, Shenyang, China

**Keywords:** granulomatous mastitis, clinical characteristic, microbiota analysis, *corynebacterium*, *corynebacterium kroppenstedtii*

## Abstract

**Introduction:**

Granulomatous mastitis (GM) is a chronic inflammatory breast disease. In recent years, the role of *Corynebacterium* in GM onset has received more and more attention. This study aims to detect the dominant bacterium in GM patients and analyze the association between clinical characteristics and infectious factors.

**Methods:**

In this study, 88 samples from 44 GM patients, six acute lactation mastitis (ALM) patients, and 25 non-inflammatory breast disease (NIB) patients were divided into a GM pus group, a GM tissue group, an ALM pus group, and a NIB tissue group; then, 16S ribosomal DNA sequencing was used to explore their microbiota. The clinical data of all 44 GM patients were also retrospectively collected and analyzed to determine their relationship with infection.

**Results:**

The median age of the 44 GM patients was 33 years, and 88.6% of patients had primary-onset cases, while 11.4% were recurrences; additionally, 89.5% of patients were postpartum and 10.5% were nulliparous. The serum prolactin level was abnormal in nine patients (24.3%). Samples from 15 GM patients (34.1%) had a *Corynebacterium* abundance of >1% (1.08–80.08%), with eight (53.3%) displaying an abundance of >10%. *Corynebacterium* was the only genus with significant differences between the GM pus group and the other three groups (*p* < 0.05). *Corynebacterium kroppenstedtii* was the predominant *Corynebacterium* species. Among clinical characteristics, a statistical difference in breast abscess formation was observed according to *Corynebacterium* abundance in *Corynebacterium*-positive and- negative patients (*p* < 0.05).

**Discussion:**

This study explored the relationship between *Corynebacterium* infection and GM, compared the clinical characteristics between *Corynebacterium*-positive and- negative patients, and provided support for the role of *Corynebacterium* species-in particular, *C. kroppenstedtii*-in the pathogenesis of GM. The detection of *Corynebacterium* can predict GM onset, especially in patients with high prolactin levels or a history of recent lactation.

## Introduction

Granulomatous mastitis (GM), a type of non-lactation mastitis, is a benign chronic inflammatory breast disease that commonly affects women of reproductive age with a history of gestation and lactation (Fujii et al., 2018). In recent years, the incidence of GM has increased rapidly (Bacon et al., 2021; Bi et al., 2021). Clinical manifestations of GM and include breast lumps, abscesses, fistulae, and nipple retractions. Sometimes, GM mimics breast carcinoma (Dobinson et al., 2015). Some patients present with extramammary symptoms, such as erythematous nodosum and joint pain, especially in the lower limbs (Saydam et al., 2020). The golden diagnostic standard of GM is pathological examination (Johnstone et al., 2017). Typical morphology under the microscope includes lobulocentric granulomatous inflammation, non-caseating granulomas, and neutrophils (Yu et al., 2016; Johnstone et al., 2017; Bi et al., 2021).

Currently, the etiology of GM remains hypothetical; however, relevant factors reported to date include immune dysregulation, microbial infection, and hormonal disturbances (Fujii et al., 2018; Koksal et al., 2020; Yin et al., 2021). In recent years, infection-related factors have received increasing attention. *Corynebacterium* species, particularly *Corynebacterium kroppenstedtii* (*Ck*), have been linked to GM (Tauch et al., 2016).

Nowadays, with the advent of new technologies, such as 16S ribosomal RNA, 16S ribosomal DNA (16SrDNA) sequencing, and matrix-assisted laser desorption ionization–time-of-flight mass spectrometry (Li et al., 2020), clinical microbiology laboratories can identify organisms down to both the genus and species levels more accurately and promptly (Saraiya and Corpuz, 2019). However, although emerging technologies offer excellent convenience for the detection of GM pathogens, there is still often an absence of clinical information about the bacterium, causing insufficient attention to be paid to infectious factors in the GM diagnosis and treatment process.

An agreement has not yet been reached regarding the treatment of GM (Yaghan et al., 2020). Conservative strategy involves immunosuppressants, steroids, and antibiotics, while surgery is a treatment choice available for patients showing an insufficient response to conservative therapy (Wolfrum et al., 2018; Chen et al., 2021). For *Ck*-related GM, choosing lipophilic antibiotics is very important. To avoid the development of drug resistance and obtain better efficacy, the course of antibiotics should not be short (Williams et al., 2021). Therefore, accurately identifying the bacteria species present will help the clinic tailor antibiotic use.

**Figure 1:**
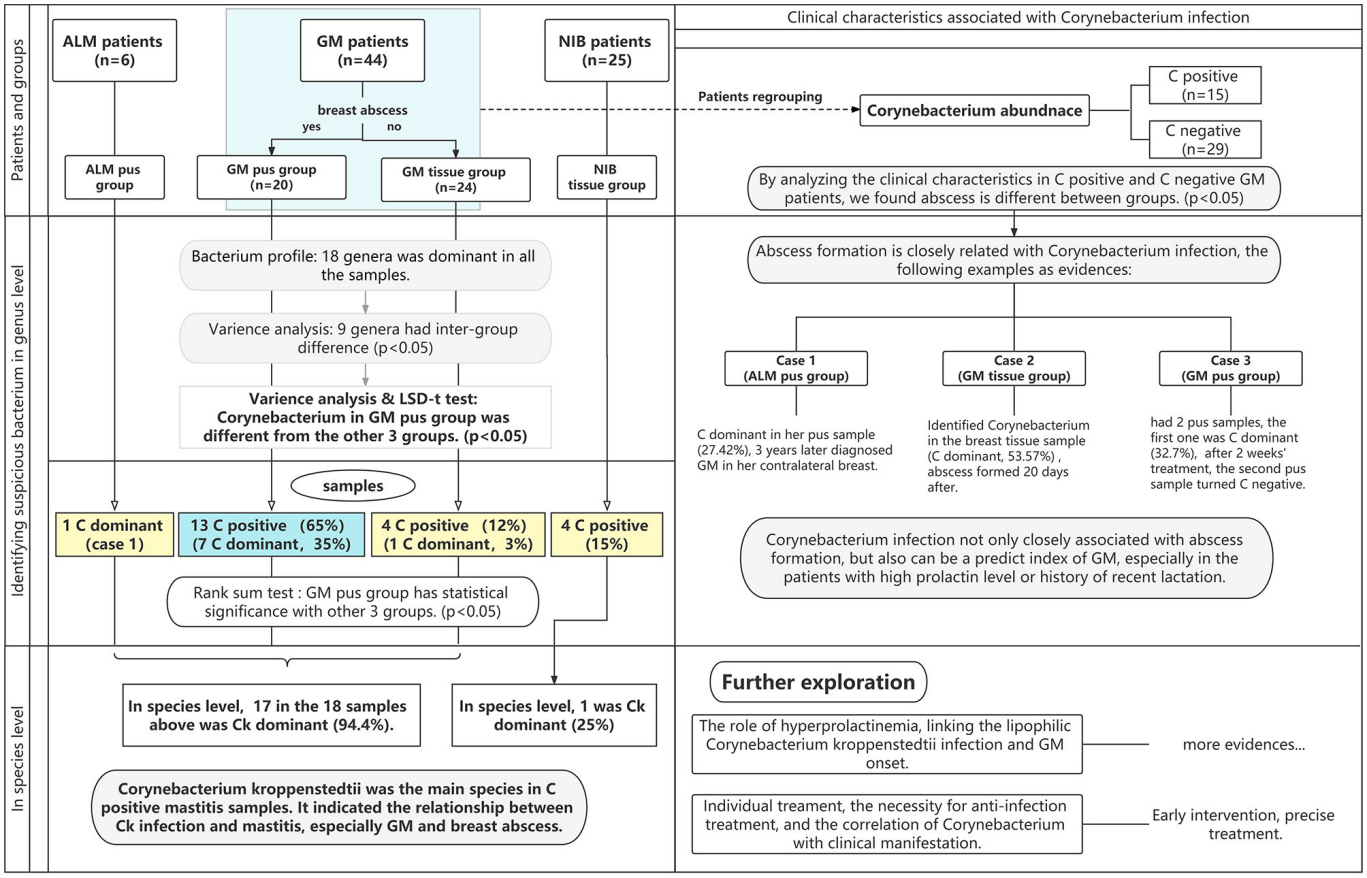
The flowchart of this study. The blue area represents the observation group, while the yellow areas represent the control group. Abbreviations: ALM, acute lactation mastitis; *Ck, Corynebacterium kroppenstedtii*; GM, granulomatous mastitis; NIB, non-inflammation breast disease. “C” is the abbreviation for *Corynebacterium*; C-dominant means the abundance of *Corynebacterium* is >10%, and C-positive means the abundance of *Corynebacterium* is >1%.

In the enrolled cases, we explored whether the identified bacteria were pathogens of GM, which bacterium was the primary pathogen of GM, the relationship between the patient's clinical manifestations and the pathogen, and the difference in bacterium abundance among different types of samples from the same patient. The microbiota in breast pus and tissue samples from GM patients were examined by 16SrDNA sequencing and compared to those of samples from patients with acute lactation mastitis (ALM) or non-inflammatory breast diseases (NIBs), including benign or malignant breast tumors. This is the first study to report bacterial differences in pus and tissue samples, and it is also the first to investigate the contrast between GM patients and ALM and NIB patients. This study aims to detect the dominant bacterium in GM patients and analyze the association between clinical characteristics and infectious factors to provide reliable evidence for early, precise treatment. Figure 1 contains a flowchart of the study selection process.

## Materials and methods

### Patients and specimens

From March 2019 to March 2022, 44 GM patients, 6 ALM patients, and 25 NIB patients were recruited for this study. GM diagnosis was proven by pathologic evidence. All enrolled patients were from the Breast department, Beijing Hospital of Traditional Chinese Medicine, Capital Medical University, and written informed consent was obtained from all participants.

**Figure 2:**
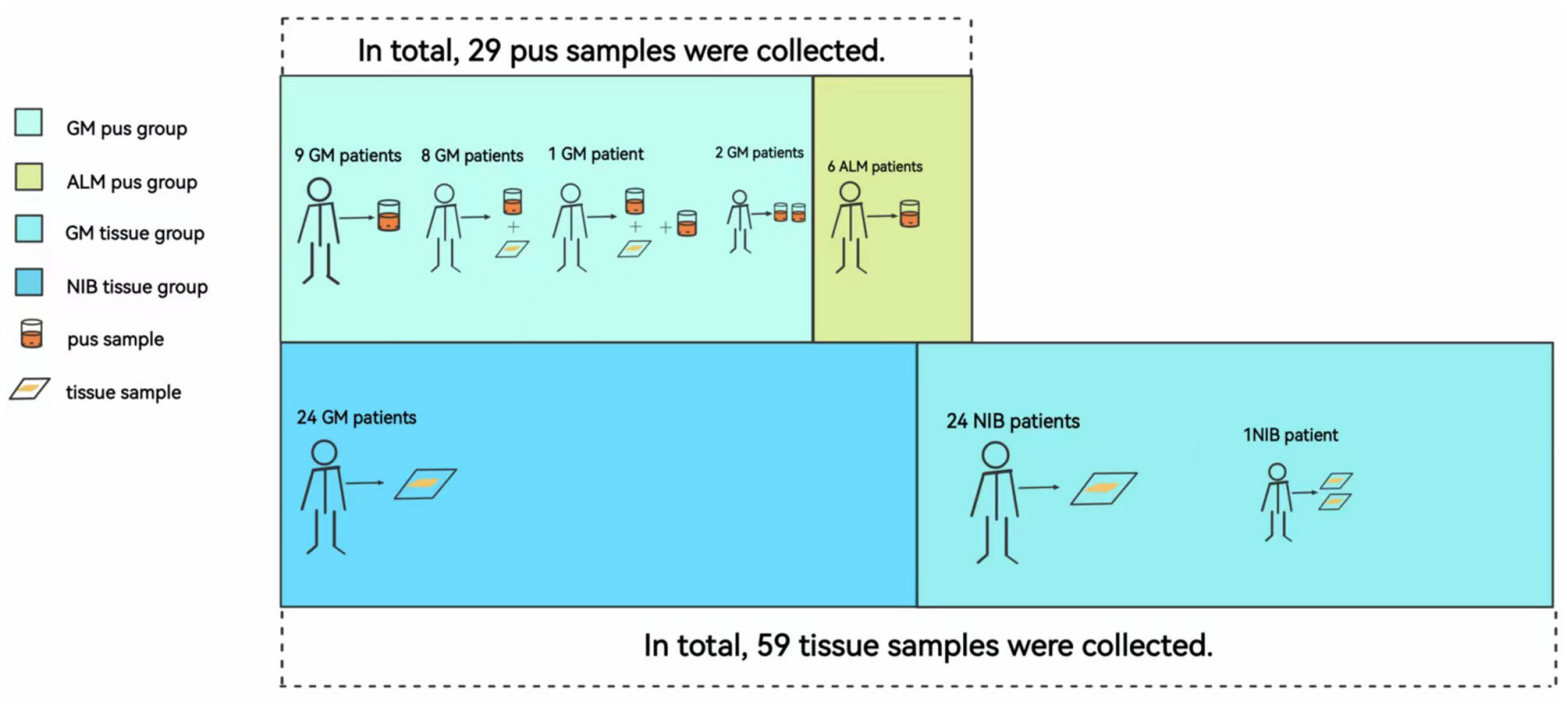
Correspondence between samples and patients in each group. Abbreviations: ALM, acute lactation mastitis; *Ck, Corynebacterium kroppenstedtii*; GM, granulomatous mastitis; NIB, non-inflammation breast disease. Patients had different samples due to their disease condition and the number of follow-up visits. In this figure, we can see that eight patients in the GM pus group had both pus and tissue samples; one patient had two pus samples taken at two different times and one tissue sample. In the NIB tissue group, one patient had two tissue samples collected because she had bilateral fibromas and underwent two surgeries at different times.

Among the 44 GM patients, 20 GM patients had breast abscesses; pus samples of all of these patients were taken, and tissue samples of nine patients were also collected. A total of 24 GM patients did not have an abscess, so only tissue samples were obtained from these individuals. All six ALM patients had breast abscesses, and we collected six pus samples from them. None of the 25 NIB patients had an abscess, so only their tissue samples were obtained. As shown in Figure 1, this study divided the patients into two pus groups (GM pus group and ALM pus group) and two tissue groups (GM tissue group and NIB tissue group). We obtained 29 pus samples and 59 tissue samples (patients with multiple samples are noted in Figure 2). During microbiota analysis, the ALM pus and NIB tissue groups were mainly used as control groups for the two GM groups.

Pus samples were obtained by syringe negative-pressure aspiration, and tissue samples were obtained from puncture or surgery. All operations followed the aseptic principle, and samples were quickly transferred to sterile containers after sampling.

Clinical data were collected for the 44 GM patients, including age, newly diagnosed or relapsed status, laterality, maternity history, clinical manifestation, serum prolactin level, and disease duration.

### 16SrDNA sequencing and bioinformatics analysis

16SrDNA sequencing was completed by Sangon Biotech (Shanghai) Co., Ltd. (Shanghai, China). The extracted genomic DNA was examined by agarose electrophoresis to check the integrity and concentration of the genomic DNA. Genomic DNA was precisely quantified using the Qubit 2.0 DNA assay kit (Thermo Fisher Scientific, Waltham, MA, United States), to determine the amount of DNA incorporated into the polymerase chain reaction (PCR). The primers used for PCR were fused to universal primers for the MiSeq sequencing platform (Illumina, San Diego, CA, United States). The PCR products were subjected to agarose electrophoresis after PCR, and the DNA was purified by using VAHTS DNA Clean Beads (Vazyme Biotech, Nanjing, China). Qubit 2.0 was used to quantify the refined products, and all samples were mixed in a 1:1 ratio according to the measured DNA concentration and shaken evenly. This mixed sample was sequenced using the MiSeq platform.

Next, the raw image data files were converted into sequenced reads by base calling analysis (Schmieder and Edwards, 2011), labeled “raw data”, and stored in a fastq (.fq) file format, which contains sequence information for sequencing sequences (reads) and their corresponding sequencing quality information.

Down-machine sequencing obtained pair-ended (PE) sequence data, and the sequencing sequence contained the barcode sequence as well as primer and adapter sequences added during sequencing. The primer adapter sequence was removed, and then, according to the overlapping relationship between PE reads, the paired reads were spliced into a sequence. Next, samples were identified and differentiated according to the barcode label sequence to obtain each sample's data. Finally, the quality of each sample's data was quality-controlled and filtered to obtain effective data for that sample.

The data-optimization methods and parameters were as follows (Marcel, 2011; Edgar, 2013): (1) we used cutadapt to remove the Read13' terminal sequencing primer linker AGATCGGAAGAGACGTCTGAACTCCAGTCA and the Read23' terminal sequencing primer linker AGATCGGAAGAGCGTGGTGTAGGGAAAGAGT; (2) we used PEAR to merge pairs of reads into a sequence according to the overlapping relationship between PE reads; (3) we split the data of each sample from the spliced data according to the barcode sequence and primer sequence of each sample, then corrected the sequence direction; and (4) we used PRINSEQ to excise bases below 20 at the tail of reads and set a window of 10 bp. If the average mass value in the window was lower than 20, we cutoff the back-end bases from the window, filtered the N-containing sequences and short sequences after quality control, and finally filtered out the sequences with low complexity.

Subsequently, OTU clustering analysis was used for each sample-optimization sequence. To be precise to the species level, BLAST was used for comparison with the GTDB database, and the community composition of each sample was finally counted at each taxonomic level as follows: domain, phylum, class, order, family, genus, and species (Altschul et al., 1997).

### Statistical analysis

SPSS version 25.0 (IBM Corporation, Armonk, NY, United States) was used for data analysis and measurement data were presented as mean ± standard deviation values while counting data were presented as numbers (percentages). STAMP version 2.1.3 was used for variance analysis to determine which variables among the many control variables had a significant impact on the observed variables. A one-way analysis of variance was performed on the top 10 flora of the four groups at the genus level, and the flora that contributed greatly to differences between groups was obtained; meanwhile, the main differentiating bacteria between the GM pus group, GM tissue group, and other groups were obtained by least significant difference multiple comparisons *post-ho*c testing. The *Corynebacterium* degree between the four groups was determined by non-parametric rank-sum testing. Finally, the clinical characteristics of GM patients between the *Corynebacterium*-positive and *Corynebacterium*-negative groups were tested by the chi-square test or exact probability method, and *p* < 0.05 was considered statistically significant.

## Results

### Characteristics of GM patients

Table 1 summarizes the clinical characteristics of the 44 patients with GM enrolled in this study. Among them, one patient had accessory axillary GM. The median age of GM patients was 33 years (range, 17–44 years), and 39 patients (88.6%) had been recently diagnosed for the first time, while five patients (11.4%) had relapsed. The interval between incipience and recurrence ranged from 3 months to 3 years, and 42 cases (95.5%) were unilateral and two (4.5%) were bilateral. According to the recorded data, four patients (10.5%) were nulliparous, while 34 patients (89.5%) were postpartum, and the median postpartum time was 3 years. The most common clinical manifestation was a palpable breast mass with an average diameter of 5.94 cm. A total of 26 patients (59.1%) had local redness, 22 patients (50%) were found to have nipple retraction, and five patients (11.4%) had nipple discharge. Serum prolactin levels were available in 37 patients, including nine patients (24.3%) with levels above the normal range. The disease duration ranged from 7 days to 1.5 years.

**Table 1:** Clinical characteristics of GM patients.

**Characteristic**	**GM Patients (*n* = 44)**
Age (median, range), years	33, 17–44
**Disease condition**	
Newly diagnosed	39
Relapsed	5
**Laterality**	
Left	21
Right	21
Bilateral	2
**Maternity history**	
Parous	34
Nulliparous	4
NR	6
Postpartum time (median, range), years	3, 0.25–15
**Clinical manifestation**	
Palpable mass	44
Diameter of the mass (average, range), cm	5.94, 1–12
Local redness	26
Nipple retraction	22
Nipple discharge	5
**Serum prolactin level**	
Normal	28
Above normal	9
NR	7
Disease duration (median, range), days	92.1, 7–545

NR, No record.

### Bacterial profiles in samples (genus level)

**Figure 3:**
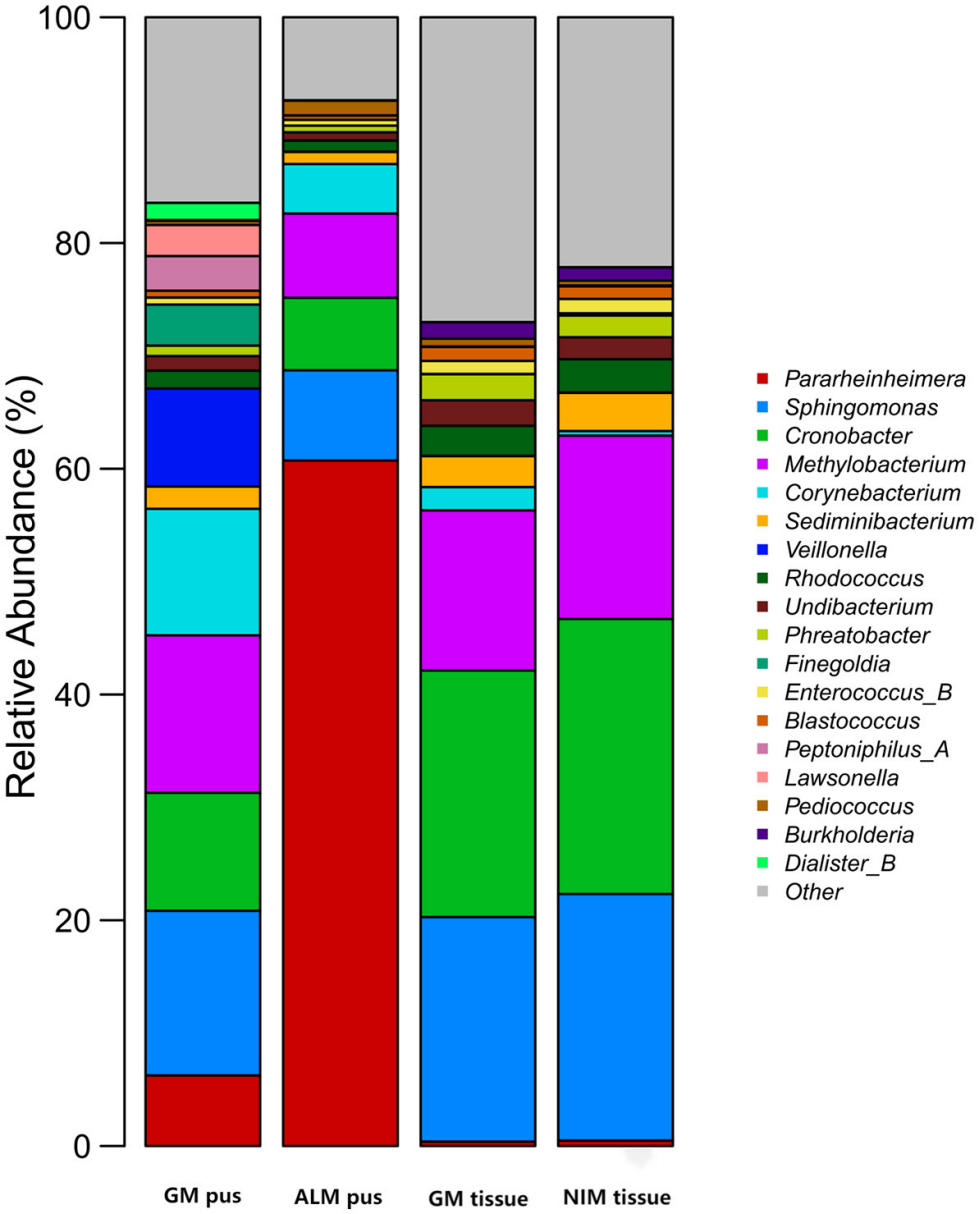
Top 10 genera in four groups. A total of 18 genera were involved.

**Figure 4:**
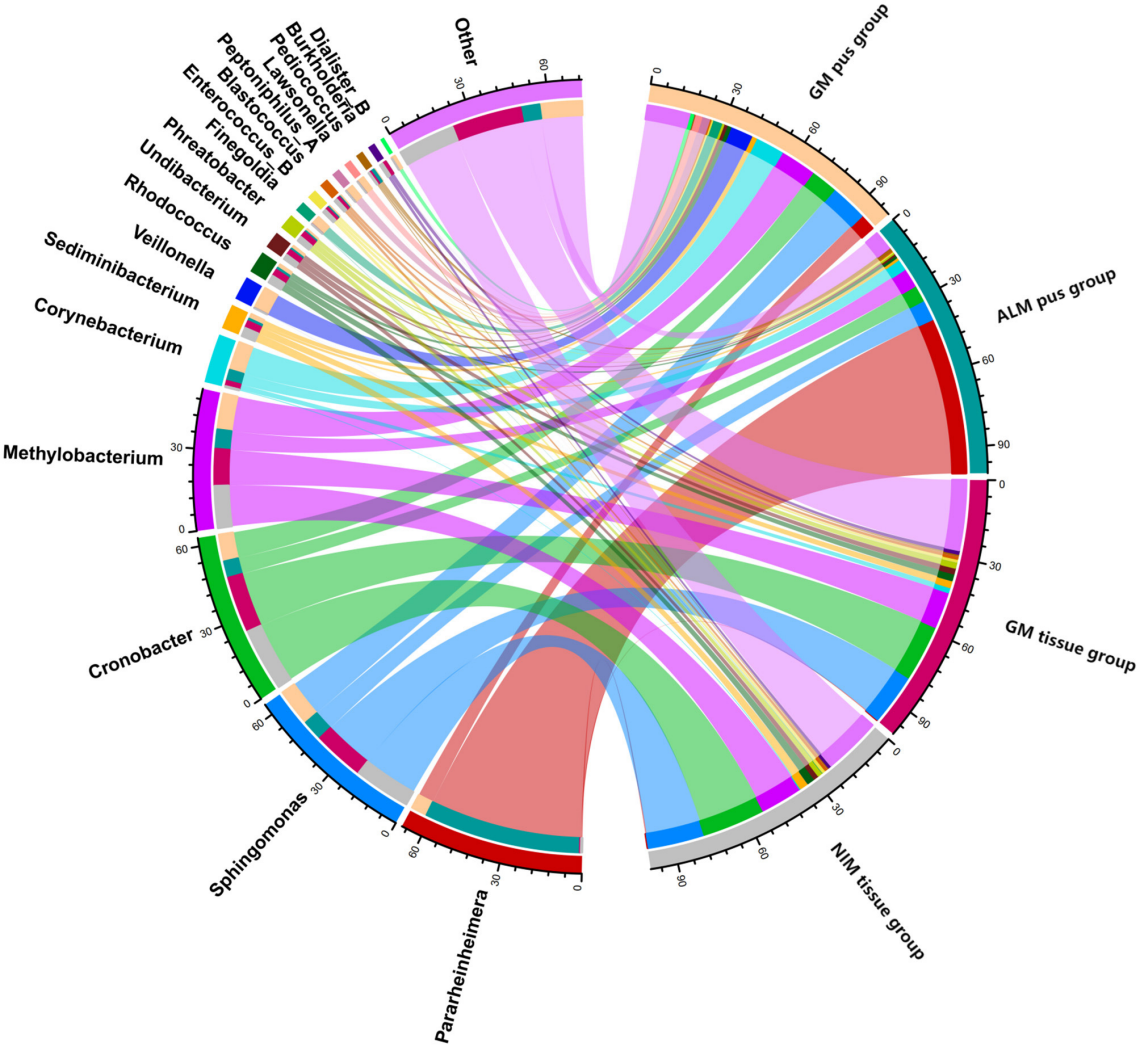
Collinearity relationship plot. The right semicircle indicates the genera abundance composition of the groups, while the left semicircle shows the proportion of genera in different groups. The number at the edge of the circle represents the genera abundance of the right semicircle and the proportion of genera on the left semicircle.

Figure 3 shows the top 10 genera in the four groups, respectively. A total of 18 genera were involved in the four groups. Figure 4 is the collinearity relationship plot, which reflects the proportion of the dominant genera composition for each group and the distribution proportion of each dominant genus between the different groups.

### Contributing bacteria

Table 2 shows the results of contributing bacteria in the four groups. All 18 genera we just mentioned were compared in these four groups, and nine genera had statistically significant differences. We further analyzed the nine genera with differences between groups, mainly focusing on the differences between the GM pus/tissue group and the other two groups. Eventually, we found that the genus *Corynebacterium* was the only bacterial genus with a significant difference between the GM pus group and the other three groups (Table 3). There was no significant difference between the GM tissue group and the two control groups (ALM pus group and NIB tissue group) (Table 4).

**Table 2:** Contributing bacteria in the four groups.

**Bacterium**	**Eta squared**	***p*-value**
Pararheinheimera	0.487	0.000^*^
Sphingomonas	0.235	0.000^*^
Cronobacter	0.346	0.000^*^
Methylobacterium	0.088	0.051
Corynebacterium	0.128	0.009^*^
Sediminibacterium	0.222	0.000^*^
Veillonella	0.066	0.124
Rhodococcus	0.233	0.000^*^
Undibacterium	0.208	0.000^*^
Phreatobacter	0.231	0.000^*^
Finegoldia	0.036	0.378
Enterococcus_B	0.029	0.474
Blastococcus	0.092	0.042^*^
Peptoniphilus_A	0.059	0.160
Lawsonella	0.032	0.430
Pediococcus	0.028	0.491
Burkholderia	0.026	0.532
Dialister_B	0.033	0.425

Eta squared: strength of the association (correlation ratio), i.e., the percentage of variation in the dependent variable explained by the independent variable. ^*^p < 0.05.

**Table 3:** Comparison between the GM pus group and the other three groups.

**Group**	**Corynebacterium abundance (%)**	***p*-value**
GM pus	11.337 ± 19.246	
ALM pus	0.028 ± 0.030	0.049^*^
GM tissue	2.060 ± 9.268	0.004^*^
NIB tissue	0.408 ± 0.493	0.001^*^

* Compared to the GM pus group, *p* < 0.05.

**Table 4:** Comparison between the GM tissue group and the other three groups.

**Group**	**Corynebacterium abundance (%)**	***p*-value**
GM tissue	2.060 ± 9.268	
ALM pus	0.028 ± 0.030	0.713
GM pus	11.337 ± 19.246	0.004^*^
NIB tissue	0.408 ± 0.493	0.584

* Compared to the GM tissue group, *p* < 0.05.

### *Corynebacterium* in samples

Since *Corynebacterium* was found to be the only contributing bacteria in the GM group, we further analyzed the abundance of *Corynebacterium* in all samples. We assigned three degrees according to the *Corynebacterium* abundance, where an abundance of 1–10% (including 1%) was “*Corynebacterium*-positive” (C-positive), an abundance of ≥10% was “*Corynebacterium*-dominant” (C-dominant), and an abundance of <1% was “*Corynebacterium*-negative” (C-negative), respectively. As Table 5 shows, six samples (26.09%) were C-positive in the GM pus group and seven samples (30.43%) were C-dominant. In total, *Corynebacterium* abundance ranged from 1.08 to 80.08%. In the ALM pus group, five samples were C-negative and another sample was excluded because the patient developed GM later (this will be discussed as case 1 in section 3.8). In the GM tissue group, three samples (9.09%) were C-positive and one sample (3.03%) was C-dominant, and the total *Corynebacterium* abundance ranged from 1.11 to 53.57% (the mentioned C-dominant sample will be discussed as case 2 in section 3.8.). In the NIB tissue group, four samples (15.38%) were C-positive, and the total *Corynebacterium* abundance ranged from 1.11 to 2.04%. As Table 5 shows, the *Corynebacterium* abundance degree in the GM pus group differed significantly from that in the other three groups.

**Table 5:** Comparison of *Corynebacterium* degree between the four groups (*n* [%]).

**Group**	***n***	**C-negative**	**C-positive**	**C-dominant**	**Z**	***p*-value**
GM pus	23	10 (43.48)	6 (26.09)	7 (30.43)		
ALM pus	6	5 (100.00)	0 (0.00)	0 (0.00)	−2.150	0.032^*^
GM tissue	33	29 (87.88)	3 (9.09)	1 (3.03)	−3.621	0.000^*^
NIB tissue	26	22 (84.62)	4 (15.38)	0 (0.00)	−3.256	0.001^*^

* Compared to the GM pus group, *p* < 0.05.

**Figure 5:**
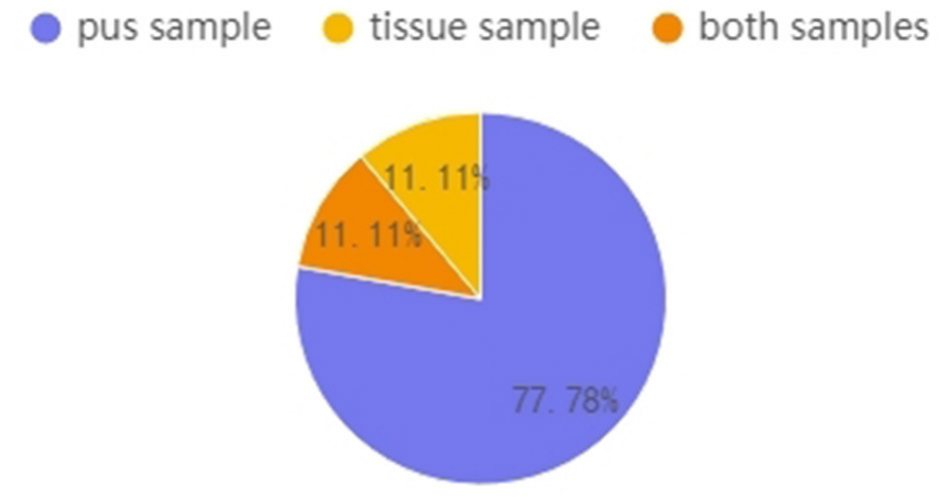
Patients who had multiple samples collected (*n* = 9). Percentages represent the proportions of C-positive and C-dominant samples from the nine GM patients.

Figure 5 shows that, in this study, nine GM patients had pus and tissue samples, and the pus samples of seven (77.78%) of these GM patients were C-positive or C-dominant. In contrast, their tissue samples were C-negative. Overall, 11.1% of patients had C-positive findings in both pus and tissue, while one patient's tissue sample was C-positive (the *Corynebacterium* abundance was 1.11%) but her pus sample was C-negative (the *Corynebacterium* abundance was 0.30%). After data tracing of this patient, we found that the tissue sample was collected before the pus sample; successive treatment may explain why the tissue result was C-positive but the pus result was C-negative.

### Different *Corynebacterium* species in samples

**Figure 6:**
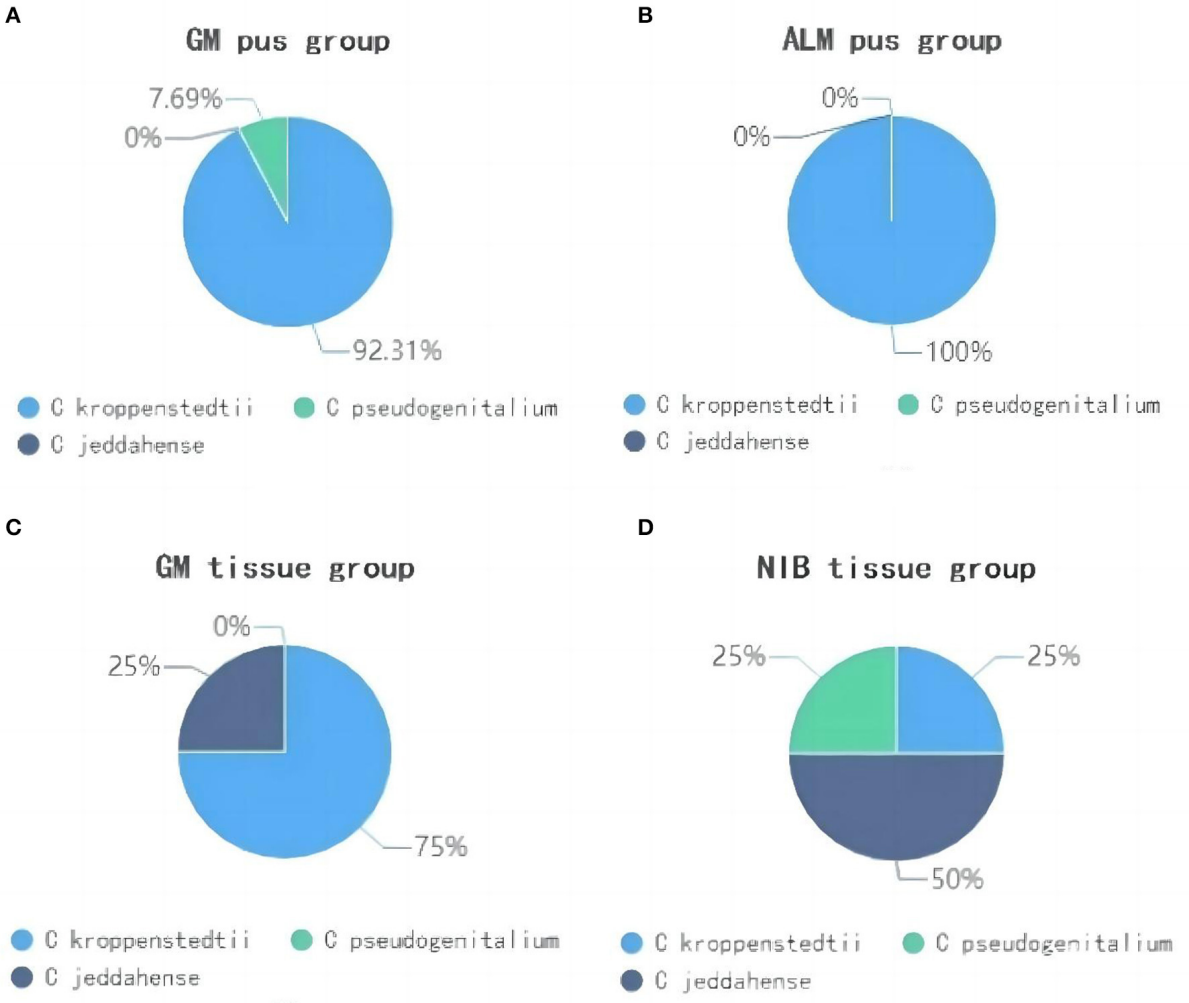
The proportion of *Corynebacterium* species in four groups. (**A**) shows the GM pus group; (**B**) shows the ALM pus group; (**C**) shows the GM tissue group; (**D**) shows the NIB tissue group.

A total of three *Corynebacterium* species were detected in this study, including *Ck, Corynebacterium jeddahense*, and *Corynebacterium pseudogenitalium*. Figure 6 shows the predominant species proportion in each group. In the GM pus group, Ck was predominant in 12 of the 13 C-positive samples (92.31%), while *C. pseudogenitalium* and *C. jeddahense* were more common in NIB tissue samples. Interestingly, one GM pus sample, in which the abundance of *Corynebacterium* was 80.08%—the highest—had a predominance of *C. pseudogenitalium*.

### Clinical characteristics associated with *Corynebacterium* infection

To explore the relationship between *Corynebacterium* infection and clinical characteristics, we divided the patients into two groups, *Corynebacterium*-positive (C-positive, where the abundance was ≥1%) and *Corynebacterium*-negative (C-negative, where the abundance was <1%). Under this delineation, 15 patients were C-positive and 29 patients were C-negative. We compared their clinical characteristics and found breast abscess was the only one that showed a significant difference between the two groups (*p* < 0.05) (Table 6).

**Table 6:** Clinical characteristics analyses.

		**C-positive (*n* = 15)**	**C-negative (*n* = 29)**	**χ^2^**	***P-*value**
Laterality	Unilateral	15	27	1.084	0.298
	Bilateral	0	2		
Breast abscess	Yes	13	7	15.590	0.000^*^
	No	2	22		
Local redness	Yes	11	15	0.224	0.636
	No	3	6		
	NR	1	8		
Disease condition	Incipience	13	26	0.088	0.767
	Relapse	2	3		
Disease duration, days	≤ 30	5	10	0.008	0.927
	>30	8	15		
	NR	2	4		
Maternity history	Nulliparous	0	4	2.608	0.106
	Parous	14	20		
	NR	1	5		
Nipple retraction	Yes	8	14	0.101	0.75
	No	7	15		

NR: no record.

* *p* < 0.05.

### Examples of typical cases

**Figure 7:**
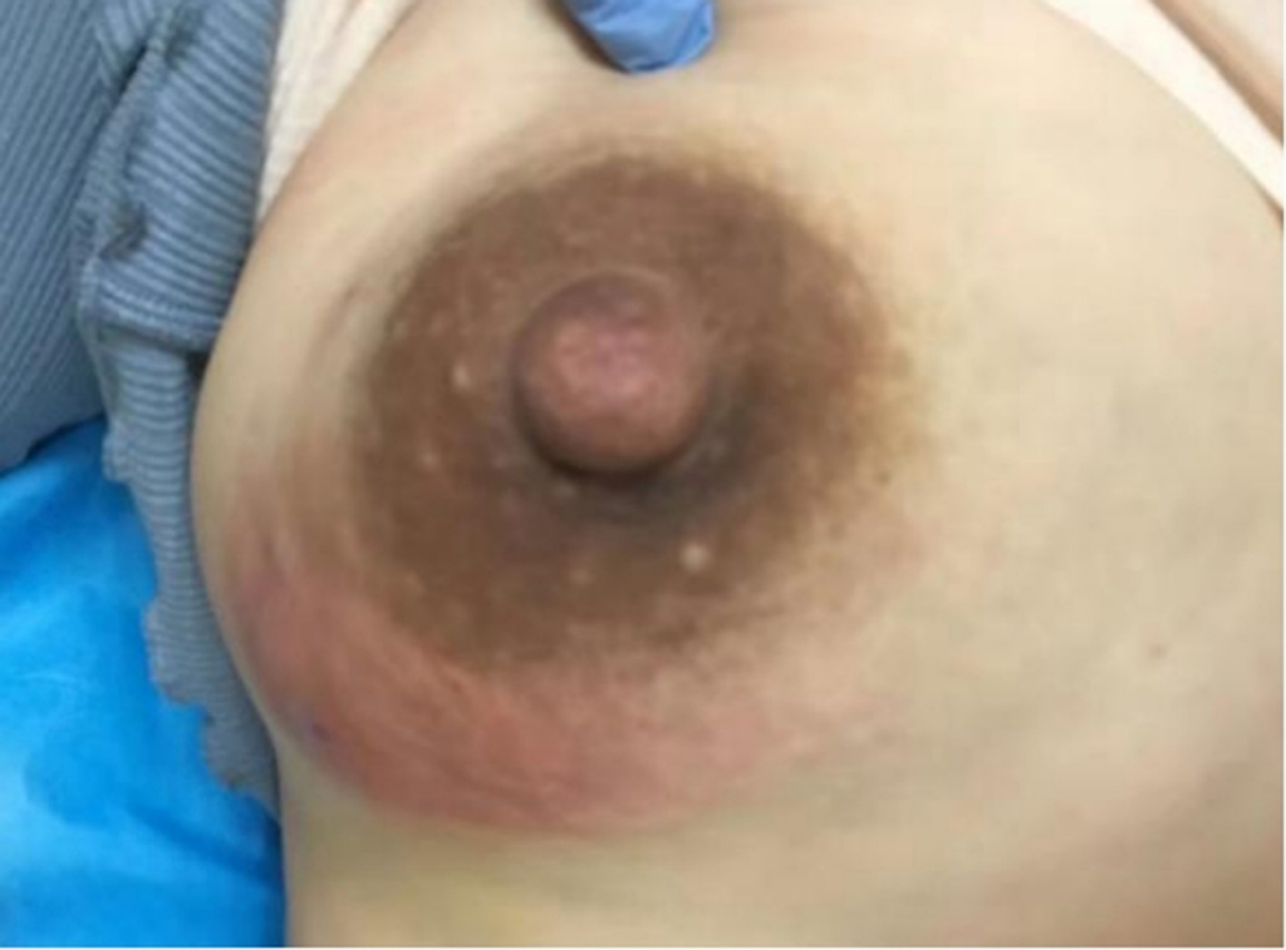
Case 1. A lesion of an ALM pus group patient is seen. The lower quadrant of the right breast was red and swollen.

Case 1 was a patient in the ALM pus group. She visited our department 2 weeks after giving birth to her second child. Painful redness and a swollen lump had been present in her right breast for a week (Figure 7), and the diagnosis was ALM. Since a breast abscess had formed, we aspirated the pus by syringe to reduce the abscess tension. The pus sample was tested, and the result was C-dominance. Her symptoms improved after treatment, and the lump in her right breast eventually disappeared. However, 3 years later, a new mass had developed in the left breast, and a core needle biopsy confirmed it was GM.

Case 2 was a C-dominant patient in the GM tissue group. She visited our department in the acute stage with a 1-week disease history, complaining of mild red swelling and a mass in her left breast. We performed a core needle biopsy to diagnose, and the tissue sample showed C-dominance with an abundance of 53.57%. Since no anti-infection treatment was administered, after 20 days, GM developed, and an abscess formed.

**Figure 8:**
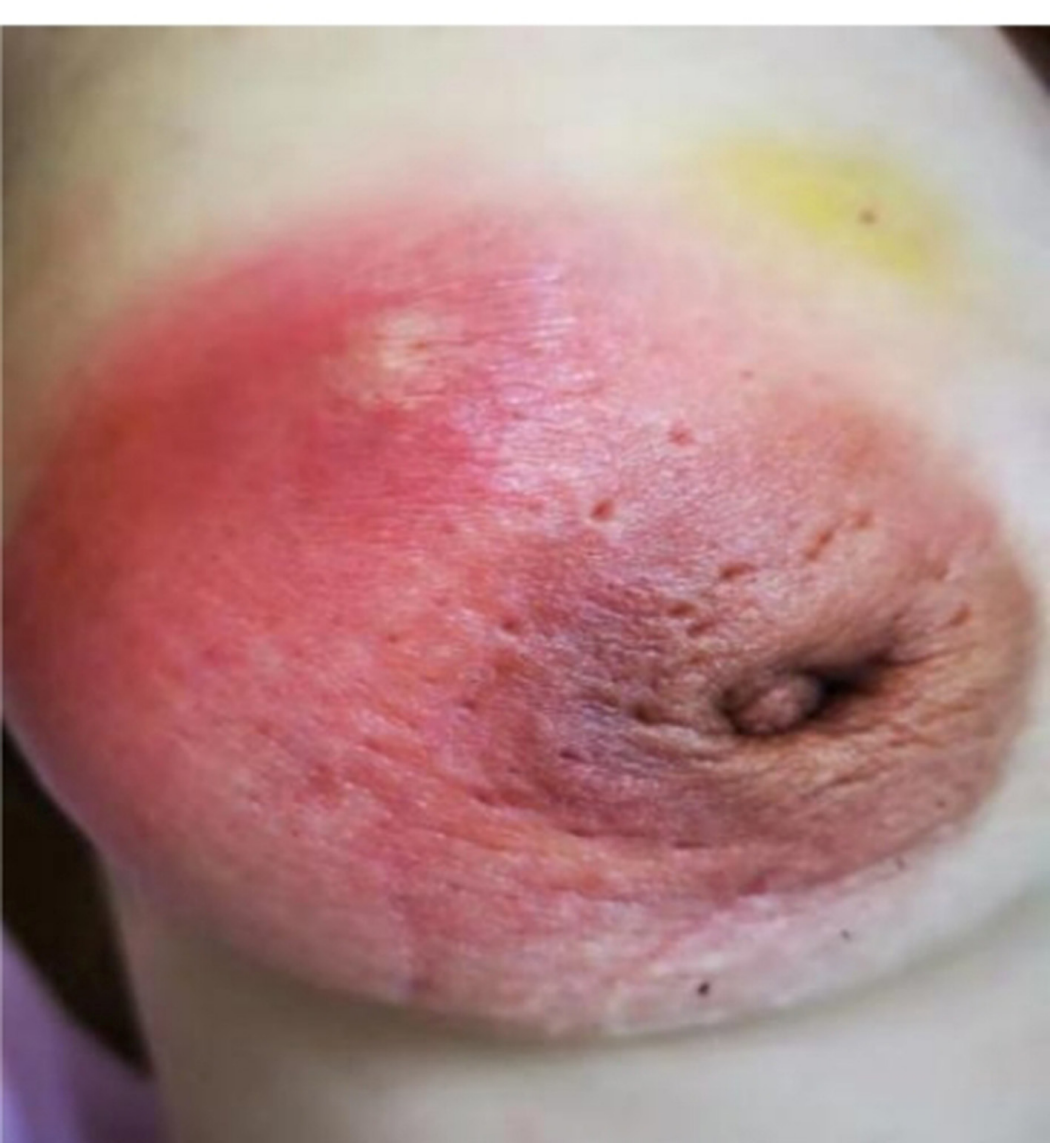
Case 2. A lesion of a GM pus group patient photographed during her initial visit is seen. The diameter of the lump was ~7 cm, and red swelling was present around the areola, with abscess formation.

Case 3 had a 1-month history of a painful right breast lump before her initial visit to our department (Figure 8). A physical examination indicated bilateral nipple reversal, left nipple discharge, and red swelling around the areola. The diameter of the lump was ~7 cm, and an abscess had formed. The pus was aspirated, and an anti-infection treatment was introduced to relieve her symptoms. After 2 weeks, her pain had decreased, and the mass was smaller in size than before. We repeated aspiration in the residue abscess and compared the pus samples obtained these two times. A considerable difference was found as follows: in the first pus sample, the abundance of *Corynebacterium* was 32.74%, while, in the second one, it was only 0.91%.

## Discussion

The theory that the “normal breast is a non-sterile environment” has already become a consensus (Saraiya and Corpuz, 2019), and diverse bacteria keep their balance in the breast microenvironment. In recent years, infectious factors have gained increasing attention in the pathogenesis of GM. Though there were some studies that focused on the relationship between infection and GM, especially *Corynebacterium* species (Dobinson et al., 2015; Wolfrum et al., 2018; Tan et al., 2020), whether this bacterium plays an essential role in the onset of GM and the relationship between bacterial infection and clinical manifestation is still not clear. Our study divided GM samples into two groups (pus and tissue groups) according to the sample type and set two control groups (an ALM pus group and a NIB tissue group) to compare the microbiota between the different groups. To the best of our knowledge, this is the first study to compare GM samples with both ALM pus samples and NIB tissue samples, rather than samples of the normal breast or another type of mastitis only, in order to identify the pathogen in GM. We also clarified the difference in pathogen abundance in GM tissue and pus, and the results offer great value for samples chosen in the microbe study of GM. The relationship between bacterial infection, clinical manifestation, and the prognosis was explored too.

Granulomatous mastitis (GM) usually occurs in women of reproductive age, ~2–6 years after delivery (Angelopoulou et al., 2018; Azizi et al., 2020; Wu and Turashvili, 2020). In this study, the average age of patients was 33 years, and the median postpartum time was 3 years. In total, 89.5% of patients were within 5 years of childbearing, and 24.3% had elevated serum prolactin levels. All these data show that GM is correlated with excessive duct secretion. Milk stasis can exist for several years after weaning, and elevated prolactin levels can stimulate ducts to oversecrete (Nikolaev et al., 2016; Bi et al., 2021; Yin et al., 2021). This can induce mammary duct dilation and cause chronic inflammation and local immune response. Moreover, the residual milk in the mammary duct provides an excellent growth environment for the lipophilic *Corynebacterium* organism.

In this study, we compared different genera in four groups and identified nine genera, such as *Pararheinheimera, Sphingomonas, Cronobacter, Corynebacterium, Sediminibacterium, Rhodococcus, Undibacterium, Phreatobacter*, and *Blastococcus*. We further performed pairwise comparisons between the four groups and found that *Corynebacterium* was the only bacterium distinguishable in the GM group; notably, its abundance in GM patients was revealed to be relatively higher by comparing with ALM and NIB patients.

*Corynebacterium* is also detectable in normal breast tissues. Due to this, whether *Corynebacterium* is a colonizer, contaminant, or pathogen has been debated for years (Wolfrum et al., 2018; Wong et al., 2018). In normal breast tissue, however, the *Corynebacterium* abundance is generally <0.5% (Li et al., 2020). Based on this premise, in this study, we chose an abundance of *Corynebacterium* of ≥1% to indicate its pathogenic nature. We divided the samples into three groups (C-negative, C-positive, and C-dominant) and used 1% and 10% as the cutoff points to delineate them. A total of 15 of 44 GM patients (34.1%) were C-positive or C-dominant, with the abundance ranging from 1.08 to 80.08%, which is significantly higher than that in the ALM and NIB groups.

At the species level, we found three *Corynebacterium* species, such as *Ck, C. jeddahense*, and *C. pseudogenitalium*. Among these, *Ck* was the predominant species in 88.2% of C-positive and C-dominant GM samples. Other studies have also identified *Ck* as the dominant species in samples. Yu et al. conducted a metagenomic analysis of 19 GM patients and found that *Ck* was predominant in 11 patients (Yu et al., 2016), while Bi et al. performed next-generation sequencing on 25 GM patients' samples and detected *Ck* in 40% (Bi et al., 2021).

Though the *Corynebacterium* abundance in the GM group was higher than that in the ALM pus and NIB tissue groups, not all GM samples were C-positive. To explore the clinical differences between samples, we also divided the GM patients into two groups, C-positive and C-negative, and compared their clinical characteristics. Laterality, local redness, nipple retraction, maternity history, and recurrence were compared, and we found that breast abscess displayed the only significant difference between the two groups. C-positive patients, especially C-dominant patients, are more likely to form abscesses, which has been proven by other research (Saraiya and Corpuz, 2019). GM recurrence related to *Corynebacterium* colonization has been reported in other studies (Le Flèche-Matéos et al., 2012; Tan et al., 2019, 2020). However, this study did not find a significant difference in recurrence between the two groups, maybe because the number of relapsed patients was too small.

Since *Corynebacterium*-related GM is more likely to result in abscess formation, it is easy to understand why this bacterium is always more abundant in pus samples than in tissue samples. Some studies tested pus samples together with tissue samples (Johnstone et al., 2017) or tested the tissue samples only (Fujii et al., 2018; Tariq et al., 2022) and rarely compared the difference between the two sample types. In this study, 56.5% of GM pus samples were C-positive, while only 8.3% of GM tissue samples were C-positive. To confirm this opinion that pus samples always have a greater *Corynebacterium* abundance than tissue samples, we analyzed nine patients with both pus and tissue samples available and found that eight patients' pus samples were C-positive, while their tissue samples generally had a lower *Corynebacterium* abundance. This result validated the view that *Corynebacterium* is prone to driving abscess formation, thus, it is more easily found in pus than in tissue.

The presence of *Corynebacterium* in pus can also be a predicting signal of GM onset. Case 1 in this study was a mother who, 2 weeks after delivery of her second child, presented with acute lactation mastitis with abscess formation in the right breast. Unlike the other lactating women whose pus samples were *Staphylococcus*-dominant, this patient's pus sample was C-dominant, and her left breast was diagnosed with GM later.

Since *Corynebacterium* is a pathogen of GM, controlling this pathogen is necessary for treatment success. If we can administer anti-infection therapy promptly, the therapeutic effect should be good and vice versa. Cases 2 and 3 are two opposing examples of GM treatment success. Case 2 had a 1-week history of a breast lump with red swelling and pain at the first visit to our department. The *Corynebacterium* abundance in her tissue sample was 53.57%, but no anti-infection treatment had been given, and a breast abscess formed 20 days later. Case 3 presented with a breast abscess and severe clinical manifestation on her first visit. Her first pus sample was C-dominant (32.47%). After 2 weeks of anti-infection treatment, her symptoms were much relieved, and the *Corynebacterium* abundance in the second sample was reduced to <1%. These two cases validate the idea that timely anti-infection treatment is effective and necessary for inhibiting *Corynebacterium* (Wolfrum et al., 2018; Yaghan et al., 2020). As *Corynebacterium* abundance decreases, the symptoms will also resolve (Johnstone et al., 2017; Zeng et al., 2022).

Our study has several limitations. First, as a retrospective study, the absence of some clinical data impacted the analysis, and bias was difficult to avoid. Second, the sample size was relatively small, especially that of the ALM pus control group. Third, due to the limited number of recurrences, the relationship between recurrence and *Corynebacterium* infection was not well defined. This will be further assessed in a future study.

## Conclusion

This study supports the correlation of *Corynebacterium* species with GM pathogenesis and identified *Ck* as the main species in GM onset. We found that *Corynebacterium* abundance in the GM group was obviously greater than that in other groups, and the abundance of *Corynebacterium* in GM pus samples was more than that in GM tissue samples. We also found that abscess formation is closely related to *Corynebacterium* infection, and early detection of *Corynebacterium* in a tissue sample may be a sign of a higher risk of abscess formation. *Corynebacterium* infection can also be a predictor of GM, especially in patients with high prolactin levels or a history of recent lactation. Timely application of anti-infection therapy is necessary for disease remission.

## Data availability statement

The data presented in the study are deposited in the NCBI repository, accession number PRJNA947767.

## Ethics statement

Written informed consent was obtained from the individual(s) for the publication of any potentially identifiable images or data included in this article.

## Author contributions

DZ and QH contributed to the conception and design of the study. WC, YZ, JY, JW, and SL collected and analyzed the data from previous studies. WC wrote the first draft of the manuscript. DZ, JC, and KM revised the manuscript. All authors contributed to manuscript revision, read, and approved the submitted version.
